# Structural basis of LARP1-mediated regulation of TOP mRNA via the 40S small ribosomal subunit

**DOI:** 10.1063/4.0000947

**Published:** 2025-10-27

**Authors:** Wenzhao Dong, Mario R. Blanco, Ross Kaufhold, Erica Wolin, Jimmy K. Guo, Marko Jovanovic, Mitchell Guttman, Jailson Brito Querido

**Affiliations:** 1 Department of Biological Chemistry, University of Michigan, Ann Arbor, MI, USA; 2 Life Sciences Institute, University of Michigan, Ann Arbor, MI, USA; 3 Division of Biology and Bioengineering, California Institute of Technology, Pasadena, CA, USA; 4 Department of Biological Sciences, Columbia University, New York, NY, USA

## Abstract

The regulation of mRNA translation is a fundamental aspect of gene expression control. Although cells have precise mechanisms to regulate each step of translation, the primary regulatory events occur during initiation. Among the transcripts subject to tight translational control are those containing a terminal oligopyrimidine (TOP) motif. In mammals, TOP mRNAs predominantly encode components of the translational machinery, including all ribosomal proteins and translation factors. La-related protein 1 (LARP1) has emerged as a key regulator of TOP mRNAs. LARP1 binds directly to the TOP motif, modulating the stability and translational activity of these transcripts. However, the molecular mechanism by which LARP1 controls translation initiation remains unresolved, with evidence supporting both translational repression and activation. Here, we used single-particle cryo-electron microscopy (cryo-EM) to identify the precise binding site of LARP1 at the mRNA entry channel in the 40S small ribosome subunit. Strikingly, the position of LARP1 within the mRNA channel is sterically incompatible with active translation, as it would clash with both incoming mRNA and initiator tRNA_i_^Met^ during translation initiation. These findings support a model in which non-translating ribosomes associate with LARP1-bound TOP mRNAs, linking TOP mRNA translation to ribosome availability. This interaction may constitute a ribosome- autoregulatory feedback loop that adjusts ribosome biogenesis in response to cellular demands.

